# Supplementary material for: Determinants of stroke among adult hypertension patients in dessie comprehensive specialized hospital and Kombolcha general hospital, Amhara, Ethiopia: A case-control study
Source: PLoS One. 2025 Apr 1;20(4):e0319016. doi: 10.1371/journal.pone.0319016 (PMC11960888; doi:10.1371/journal.pone.0319016)
Supplement: S1 File — (DOCX) [file pone.0319016.s001.docx]

**Supporting Information**

**Figure S1.**  **Schematic presentation of the sampling technique**

**Table S1. Sample size calculation with double population proportion formula using Epi-info version 7 for to identify the determinants of stroke among adult patients in DCSH and kombolcha general hospital, North eastern Ethiopia.**

**Table S2. Socio-demographic characteristics of adult stroke patients, 2023 (N=525)**

**Table S3. Lifestyle and behavioral factors of adult stroke patients, 2023 (N=525)**

**Table S4. Comorbid illnesses of adult stroke patients, 2023 (N=525)**

**Table S5. Dietary factors of adult stroke patients, 2023 (N=525)**

**Table S6. Bivariate and multivariable logistic regression model predicting the odds of stroke among adult patients, 2023 (N=525)**

**Figure S1.** **Schematic presentation of the sampling technique**

**Table S1.** Sample size calculation with double population proportion formula using Epi-info version 7 for to identify the determinants of stroke among adult patients in DCSH and kombolcha general hospital, North eastern Ethiopia(1,6,9,21).

| Factors | Percent of controls exposed | Percent of cases with exposure | AOR | sample size | 10% non-response rate | Total sample size |
| --- | --- | --- | --- | --- | --- | --- |
| Lost follow-up | 12.9 | 36 | 3.79 | 134 | 13 | 147 |
| Drinking alcohol | 13.8 | 36 | 3.51 | 146 | 15 | 161 |
| Eating fatty foods | 27.9 | 41.2 | 1.81 | 477 | 48 | 525 |
| No medication adherence | 29.8 | 66.3 | 4.6 | 75 | 8 | 83 |
| Uncontrolled diastolic BP | 54.2 | 79.8 | 3.34 | 137 | 14 | 151 |
| Having DM | 2.3 | 18.9 | 3.79 | 134 | 13 | 147 |
| Cigarette smoking | 2.6 | 15.5 | 3.51 | 146 | 15 | 161 |
| Lost follow-up | 12.9 | 36 | 1.81 | 477 | 48 | 525 |

**Table S2:** Socio-demographic characteristics of adult stroke patients, 2023 (N=525)

| Variables | Category | Cases (%) | Control (%) |
| --- | --- | --- | --- |
| Age | 18-27 years | 11 (10.9) | 59 (14) |
|  | 28-37 years | 32 (30) | 127 (30) |
|  | Above 37 years | 62 (58.9) | 234 (56) |
| Sex of respondents | Female | 43(41.1) | 140 (33.4) |
|  | Male | 62 (58.9) | 280 (66.6) |
| Residence | Urban | 85 (80.9) | 123 (29.4) |
|  | Rural | 20 (19.1) | 297 (70.4) |
| Religion | Orthodox | 36 (34.3) | 157 (37.4) |
|  | Muslim | 51(48.5) | 201 (48) |
|  | Protestant | 13(12) | 34(8) |
|  | Catholic | 5(4.7) | 28 (6.6) |
| Educational status | Illiterate | 24 (22.9) | 87 (20.6) |
|  | Able to read& write | 31 (29.7) | 128 (30.6) |
|  | Primary school | 23 (21.7) | 87 (20.6) |
|  | Secondary school | 16 (14.9) | 60 (14.3) |
|  | College/university | 11 (10.9) | 59 (14) |
| Marital status | Married | 49 (46.3) | 200 (47.7) |
|  | Single | 21 (20) | 80 (18.9) |
|  | Divorced | 23 (22.3) | 91 (21.7) |
|  | Widowed | 12 (11.4) | 49 (11.7) |
| Current occupation | Farmer | 15 (14.3) | 73 (17.4) |
|  | Merchant | 24(22.8) | 115(27.4) |
|  | Governmental employee | 29 (27.6) | 107 (25.5) |
|  | Non-government employee | 21 (20) | 82 (19.5) |
|  | Others | 16 (15.3) | 43 (10.2) |
| Distance of HF | Less than 30 km | 5 (4.7) | 34 (8) |
|  | 30-60 km | 14 (13.3) | 159 (38) |
|  | Above 60 km | 86 (82) | 227 (54) |

**Table S3:** Lifestyle and behavioral factors of adult stroke patients, 2023 (N=525)

| Variables (n=525) | Category | Cases (%) | Control (%) |
| --- | --- | --- | --- |
| Smoking status | Currently smoke | 53 (50.9) | 81(19.4) |
|  | Former smoker | 6 (5.7) | 77 (18.3) |
|  | Never smoke | 46 (43.4) | 262 (62.3) |
| Average frequency of smoking cigarettes | =< 3 times | 52 (49.7) | 315 (75.1) |
|  | >3 times | 53(50.3) | 105 (24.9) |
| Monthly income | <1500 ETB | 19 (18.3) | 77(18.3) |
|  | 1500-3000 ETB | 32 (31.4) | 132 (31.4) |
|  | 3000-4500 ETB | 19 (18.3) | 119 (28.3) |
|  | >= 4500 ETB | 35 (32) | 92(22) |
| Drinking status | Currently, drink | 62 (59.4) | 120 (28.6) |
|  | Formerly drink | 21 (20) | 107 (25.4) |
|  | Never drinking | 22 (20.6) | 193 (46) |
| Type of transport used going to work | On foot | 62 (59.4) | 256(60.9) |
|  | Ride a bicycle/motor | 17 (16) | 63 (15.1) |
|  | By car/taxi | 26 (24.6) | 101 (24) |
| Having a family history of hypertension | Yes | 64 (61.1) | 158 (37.7) |
|  | No | 41(38.9) | 262 (62.3) |
| Having information on their current BMI | Yes | 31 (29.9) | 162 (38.6) |
|  | No | 74 (70.1) | 258(61.4) |
| Current BMI status | <18 kg/m2 | 19 (17.7) | 73 (17.4) |
|  | 18-24.9 kg/m2 | 50 (48) | 204(48.6) |
|  | >= 25kg/m2 | 36 (34.3) | 143 (34) |
| Doing active exercise during the week | Yes | 51 (48.6) | 119 (28.3) |
|  | No | 54(51.4) | 301 (71.7) |

**Table S4:** Comorbid illnesses of adult stroke patients, 2023 (N=525)

| Variables (n=525) | Category | Cases (%) | Control (%) |
| --- | --- | --- | --- |
| Having any comorbid illness | Yes | 60(57.1 | 152 (36.3) |
|  | No | 45 (42.9) | 268 (63.7) |
| Comorbidity with DM | Yes | 46 (44) | 115 (27.4) |
|  | No | 59 (56) | 305 (72.6) |
| Duration of HTN | Less than five year | 51(48.6) | 120 (28.6) |
|  | More than five year | 54(51.4) | 300 (71.4) |
| Comorbidity with heart disease | Yes | 44 (41.7) | 103 (24.6) |
|  | No | 61 (58.3) | 317 (75.4) |

**Table S5:** Dietary factors of adult stroke patients, 2023 (N=525)

| Variables (n=525) | Category | Cases (%) | Control (%) |
| --- | --- | --- | --- |
| Consumption of fatty foods | Daily | 29 (27.4) | 86 (20.6) |
|  | Weekly | 23 (22.3) | 131 (31.1) |
|  | Monthly | 53 (50.3) | 203 (48.3) |
| Consumption of fruits | Daily | 19 (18.3) | 56(13.4) |
|  | Weekly | 16 (15.4) | 105 (24.9) |
|  | At least monthly | 70 (66.3) | 259 (61.7) |
| Consumption of salty foods | Daily | 46 (44) | 164 (39.1) |
|  | sometimes | 39 (36.6) | 148(35.1) |
|  | Never | 20 (19.4) | 108 (25.7) |
| Consumption of vegetables | Daily | 25 (24) | 54 (12.9) |
|  | Weekly | 22(21.1) | 97 (23.1) |
|  | At least monthly | 58(54.9) | 269 (64) |
| Consumption of water more than 2 liters/day | Daily | 23 (22) | 95 (22.6) |
|  | Weekly | 27 (27.5) | 115 (27.4) |
|  | At least monthly | 55 (52.5) | 210 (50) |

**Table S6:** Bivariable and multivariable logistic regression model predicting the odds of stroke among adult patients, 2023 (N=525)

| Variables | Response | Stroke | | COR 95%CI | AOR 95%CI |
| --- | --- | --- | --- | --- | --- |
|  |  | Cases | Controls |  |  |
| Smoking status | Currently smoke | 53 | 82 | 4.2(3.2-5.01) | 3.2 (2.1-5.2) ** |
|  | Former smoker | 6 | 77 | 0.26 (0.03-0.4) | 0.12 (0.03-1.3) |
|  | Never smoke | 46 | 261 | 1 | 1 |
| Drinking status | Currently, drink | 62 | 120 | 3.6 (2.9-7.3) | 4.7 (2.87-7.97) ** |
|  | Former drinker | 21 | 107 | 0.73 (0.03-1.09) | 1.97 (1.08-3.5) * |
|  | Never drink | 22 | 193 | 1 | 1 |
| Having a family history of hypertension | Yes | 64 | 158 | 2.59 (1.7-3.7) | 2.8 (1.8-4.3) * |
|  | No | 41 | 262 | 1 | 1 |
| Being physically active | Yes | 51 | 119 | 1 | 1 |
|  | No | 54 | 301 | 2.39 (1.6-3.49) | 2.13 (1.4-3.3) ** |
| Being comorbid with heart disease | Yes | 44 | 103 | 2.2 (1.49-3.23) | 2.04 (1.3-3.25) ** |
|  | No | 61 | 317 | 1 | 1 |
| Duration of hypertension | >5 year | 54 | 300 | 0.4 (0.09-0.89) | 1.96 (0.4-9.2) |
|  | ≤5 year | 51 | 120 | 1 | 1 |
| Being comorbid with DM | Yes | 46 | 115 | 2.1 (1.4-3.1) | 1.43(0.79-2.56) |
|  | No | 59 | 305 | 1 | 1 |

Note: COR (Crude Odds Ratio), AOR (Adjusted Odds Ratio); * (p < 0.05), ** (P ≤ 0.001), *** (p<0.0001), Hosmer and Lemeshow goodness of fit test= 0.824
