# Supplementary material for: Determinants of stroke among adult hypertension patients in dessie comprehensive specialized hospital and Kombolcha general hospital, Amhara, Ethiopia: A case-control study
Source: PLoS One. 2025 Apr 1;20(4):e0319016. doi: 10.1371/journal.pone.0319016 (PMC11960888; doi:10.1371/journal.pone.0319016)
Supplement: Attachment — Submitted filename: Response_to_Reviewers_auresp_2.docx [file pone.0319016.s005.docx]

Date: 03 /01/2025

Denekew Bitew Belay

PLOS ONE

Dear… Denekew Bitew Belay

Thank you for giving us the opportunity to submit a revised draft of our manuscript titled

“Determinants of Stroke among Adult Hypertension Patients in Dessie Comprehensive Specialized Hospital and Kombolcha General Hospital, Amhara, Ethiopia: A Case-Control Study” to the PLOS ONE. We appreciate the time and effort that you and the reviewers have dedicated to providing your valuable feedback on our manuscript. We are grateful to the reviewers for their insightful comments on our paper. We have been able to incorporate changes to reflect most of the suggestions provided by the reviewers. We have highlighted the changes within the manuscript.

Please let us know if you still have any questions or concerns about the manuscript. We will be happy to address them, now in a timely manner.

Sincerely,

Yonas Fissha Adem

**Point by Point Response to - Editor**

**Point by Point Response to – Reviewers**

**Additional Editor Comments:**

The manuscript still needs substantial improvements. Please pay attention and make the required revisions. The Sample size determination still needs improvements.

***Response:*** *Thank you for your suggestions. We will improve it as per your recommendations.*

**Reviewer #3: Dear Author/s**
Thank you for addressing many of the comments and making improvements in the manuscript. However, there are still some concerns that were not adequately addressed in this round of revisions.
1. The abbreviations are not correctly abbreviated eg. Medical Record Number (MRN) mentioned two times on page 8 and 9.

***Response:*** *Thank you for your suggestions. But in our health facility setting MRN stands for medical record number. If you have other idea for MRN abbreviation. Give us your idea and we can modify according to your comments.*
2. some of my questions are not replied, for example
A. my suggestions on sample size determination “Sample Size Determination: The explanation of your sample size determination is unclear. Provide a detailed description and support it with appropriate citations or sources. This will help validate your methodology and ensure that the sample size is justified based on relevant statistical or research standards. “
The Response is “Thank you so much for your careful check. But we have already explained it as much as possible even by putting a reference from previous study. But if you have a specific comment, we are happy to modify it.”
Now I can ask more questions as What is sample size determinations and what is it is purpose? Sample size determination is the process of calculating the number of participants or observations needed in a study to achieve reliable and valid results. Therefore, where is your sample size calculations? How can you determine the sample size? Your readers need clarity about your sample size determination? If your sample size determination is wrong then what will happen about work?

***Response:*** *Thank you very much dear reviewer, we have calculated the required sample size by using double population proportion formula using Epi Info 7 Software version 2.1.1. We didn’t use single population formula to get the largest sample size because this study isn’t cross-sectional study rather it’s a case control study so we calculate the sample size using factors associated with stroke from previous studies. We will show these factors using a table in the revised manuscript. You can check these using Epi info 7 software versions. Page 8 and line number 138-149*

B. My question “Data processing and analysis: What exactly does this entail? Are you referring to statistical methods, or something else? Please revise the methodology section, briefly and in detail, to clarify how you could apply bivariable and multivariable logistic regression. Since these methods are used to compare models for calculating your Crude Odds Ratio (COR) and Adjusted Odds Ratio (AOR), please also include the relevant statistical model equations in your explanation.”
The response is: “Thank you for your valuable input. We modified it accordingly in the revised manuscript.” But I could not find the modified methods in the revised manuscript.

***Response:*** *Thank you for your suggestions. Dear, l seems that l have changed very well the “Data processing and analysis part” in previous revised manuscript as per your recommendation. But you are not satisfied. I will revise again as much as I can but l am stressed how I satisfied you. Because l considered that l was write all things about logistic regression. Page 11 and line number 218-223.*
3. Under section: Study Design, Geographic Scope, and Time Frame
There is no clear evidence for your information, please put the references where did you get this information
***Response:*** *Thank you for your suggestions. We incorporated the reference for the information. page 6, line number 118*
4. In general, I could not identify where the responses to the comments are located, as there are no page or line numbers provided. Many answers are vague, using phrases like “we have done based on the comments” or “modified as per the comments,” without offering concrete evidence or detailed justifications. This lack of clarity makes it difficult to understand the rebuttals or acceptances in the manuscript.
Therefore, I strongly recommend providing clearer and more detailed explanations for the questions, including the specific page and line numbers where your responses can be found.

***Response:*** *Thank you for your suggestions. We incorporated the page number and line numbers for each comments.*
