# Supplementary material for: Determinants of stroke among adult hypertension patients in dessie comprehensive specialized hospital and Kombolcha general hospital, Amhara, Ethiopia: A case-control study
Source: PLoS One. 2025 Apr 1;20(4):e0319016. doi: 10.1371/journal.pone.0319016 (PMC11960888; doi:10.1371/journal.pone.0319016)
Supplement: Attachment — Submitted filename: Plos One Review.docx [file pone.0319016.s002.docx]

Thank you for giving me the opportunity to review the manuscript titled “***Determinants of Stroke among Adult Hypertension Patients in Dessie Comprehensive Specialized Hospital and Kombolcha General Hospital, Amhara Ethiopia: A Case-Control Study’*** I apologize for any inconvenience caused by the delay in my review.

The problem raised by the researchers and the hospital data used are impressive and make the work suitable for publication in *PLoS ONE*. However, the manuscript has several weaknesses that need addressing: Addressing the following issues will enhance the clarity and professionalism of the manuscript, making it more suitable for publication.

1. **Language and Editing**: The manuscript requires significant improvement in language editing. This includes correcting spelling and typographical errors, as well as grammatical mistakes.
2. **Abbreviations**: Ensure all abbreviations are properly defined when first introduced. For instance:

MRN (line 153), BMI (line 174), HO (line 191), UNICEF (line 194), "Patent" should be corrected to "patient" (line 258)

Generally, please make a thorough review of the document to correct these errors and ensure consistency throughout.

**Introduction**

The introduction is relatively well written

**Methods and materials**

1. **"Study Design, Area, and Period"**: The subtitle "Study Design, Area, and Period" may not clearly convey the intended content. Consider rephrasing it to better reflect the specifics of your study, such as "Study Design, Geographic Scope, and Time Frame," to make it more descriptive and accurate.
2. **Source of Information**: Clarify the source of your information, especially regarding the figure of 10 million in the DCSH catchment areas. Verify this number and cite the source accurately. If you cannot provide a source, consider removing this figure from the document to maintain credibility.
3. **Source Population vs. Study Population**: Define the difference between the source population and the study population. If your intent is to describe a specific group from which the sample is drawn, use "study population" consistently. Avoid confusion by choosing one term and using it throughout the document.
4. **Sample Size Determination**: The explanation of your sample size determination is unclear. Provide a detailed description and support it with appropriate citations or sources. This will help validate your methodology and ensure that the sample size is justified based on relevant statistical or research standards.
5. **Operational Definitions**: The operational definitions are well-written and supported with evidence. Ensure that each definition is clearly defined and referenced with appropriate sources to enhance the reliability and validity of your study.

These adjustments should help clarify and strengthen the presentation of your study's methodology and findings.

**Data quality control**

Is there a specific reason why the pre-test is being conducted at Boru Meda Hospital, which is another hospital included in your study?

**Data processing and analysis**: What exactly does this entail? Are you referring to statistical methods, or something else? Please revise the methodology section, briefly and in detail, to clarify how you could apply bivariable and multivariable logistic regression. Since these methods are used to compare models for calculating your Crude Odds Ratio (COR) and Adjusted Odds Ratio (AOR), please also include the relevant statistical model equations in your explanation.

**Results:**

In Table 1, for the "Current Occupation" category, you classify occupations as private, governmental, and NGOs. However, it's unclear where groups like unemployed individuals, farmers, or merchants fall within these classifications. Could you clarify which category they belong to or consider adding additional categories for these occupations?

Additionally, please spell out "Non-Governmental Organizations (NGOs)" when first mentioned, and avoid using "gov’tal" as it is not a proper abbreviation. Instead, use "governmental" for clarity.

The descriptive statistics provided from Table 1 to Table 4 are insightful, but they require more in-depth explanations. Ensure that each table's findings are clearly explained to highlight the important trends or patterns observed in the data.

As for Table 5, which presents the analysis of your logistic regression models, while the presentation is good, it would benefit from more detailed interpretations. Specifically, for the significant variables, please interpret the odds ratios (OR) of each category in relation to their reference groups. For example, explain how an odds ratio above or below 1 indicates increased or decreased likelihood of the outcome compared to the reference category. This will help clarify the real-world implications of the results.

**Discussion and conclusion**

The discussion section of the manuscript is relatively well written, providing a good analysis of the findings. However, the conclusion requires improvement to more effectively summarize the key results and their implications.

**Recommendations**: The recommendations should be more specific. Clearly identify the target audience for these recommendations, whether they are intended for national, regional, zonal, or woreda levels, or any other specific bodies. This specificity will help ensure that the recommendations are actionable and relevant to the appropriate stakeholders.
